# Supplementary material for: Overcoming resolution attenuation during tilted cryo-EM data collection
Source: Nat Commun. 2024 Jan 9;15:389. doi: 10.1038/s41467-023-44555-7 (PMC10776679; doi:10.1038/s41467-023-44555-7)
Supplement: Supplementary file 1 — Supplementary Information [file 41467_2023_44555_MOESM1_ESM.pdf]

Supplementary Information For

Overcoming Resolution Attenuation During Tilted Cryo-EM Data Collection

Sriram Aiyer[1], Philip R. Baldwin[1,2], Shi Min Tan[3], Zelin Shan[1], Juntaek Oh[4,5], Atousa Mehrani[1], Marianne E Bowman[6], Gordon Louie[6], Dario Oliveira Passos[1], Selena Đorđević-Marquardt[1], Mario Mietzsch[7], Joshua A. Hull[7], Shuichi Hoshika[8], Benjamin A. Barad[9], Danielle A. Grotjahn[9], Robert McKenna[7], Mavis Agbandje-McKenna[7], Steven A. Benner[8], Joseph A. P. Noel[6,10], Dong Wang[4,10,11], Yong Zi Tan[3,12,13,#], Dmitry Lyumkis[1,9,14#]

1. Laboratory of Genetics, The Salk Institute for Biological Studies, La Jolla, CA 92037, USA.
2. Department of Biochemistry and Molecular Biology, Baylor College of Medicine, Houston, TX 77030, USA.
3. Department of Biological Sciences, National University of Singapore, 16 Science Drive 4, Singapore 117558, Singapore.
4. Division of Pharmaceutical Sciences, Skaggs School of Pharmacy & Pharmaceutical Sciences, University of California, San Diego, La Jolla, CA, 92093, USA.
5. College of Pharmacy, Kyung Hee University, Seoul 02247, Republic of Korea
6. Jack H. Skirball Center for Chemical Biology and Proteomics, The Salk Institute for Biological Studies, La Jolla, CA 92037, USA.
7. Department of Biochemistry and Molecular Biology, College of Medicine, University of Florida, Gainesville, FL 32610.
8. Foundation for Applied Molecular Evolution, 13709 Progress Blvd Box 7, Alachua, FL 32615, USA.
9. Department of Integrative Structural and Computational Biology, The Scripps Research Institute, La Jolla, CA 92037, USA.
10. Department of Chemistry and Biochemistry, University of California San Diego, La Jolla, CA 92093, USA.
11. Department of Cellular and Molecular Medicine, University of California, San Diego, La Jolla, CA 92093, USA.
12. Disease Intervention Technology Laboratory (DITL), Agency for Science, Technology and Research (A*STAR), 8A Biomedical Grove, Singapore 138648, Singapore.
13. Institute of Molecular and Cell Biology (IMCB), Agency for Science, Technology and Research (A*STAR), 61 Biopolis Drive, Proteos, Singapore 138673, Republic of Singapore.
14. Graduate School of Biological Sciences, Section of Molecular Biology, University of California San Diego, La Jolla, CA 92093, USA.

[#]*For materials and correspondence*: Dmitry Lyumkis (dlyumkis@salk.edu), Yong Zi Tan (yztan@nus.edu.sg)

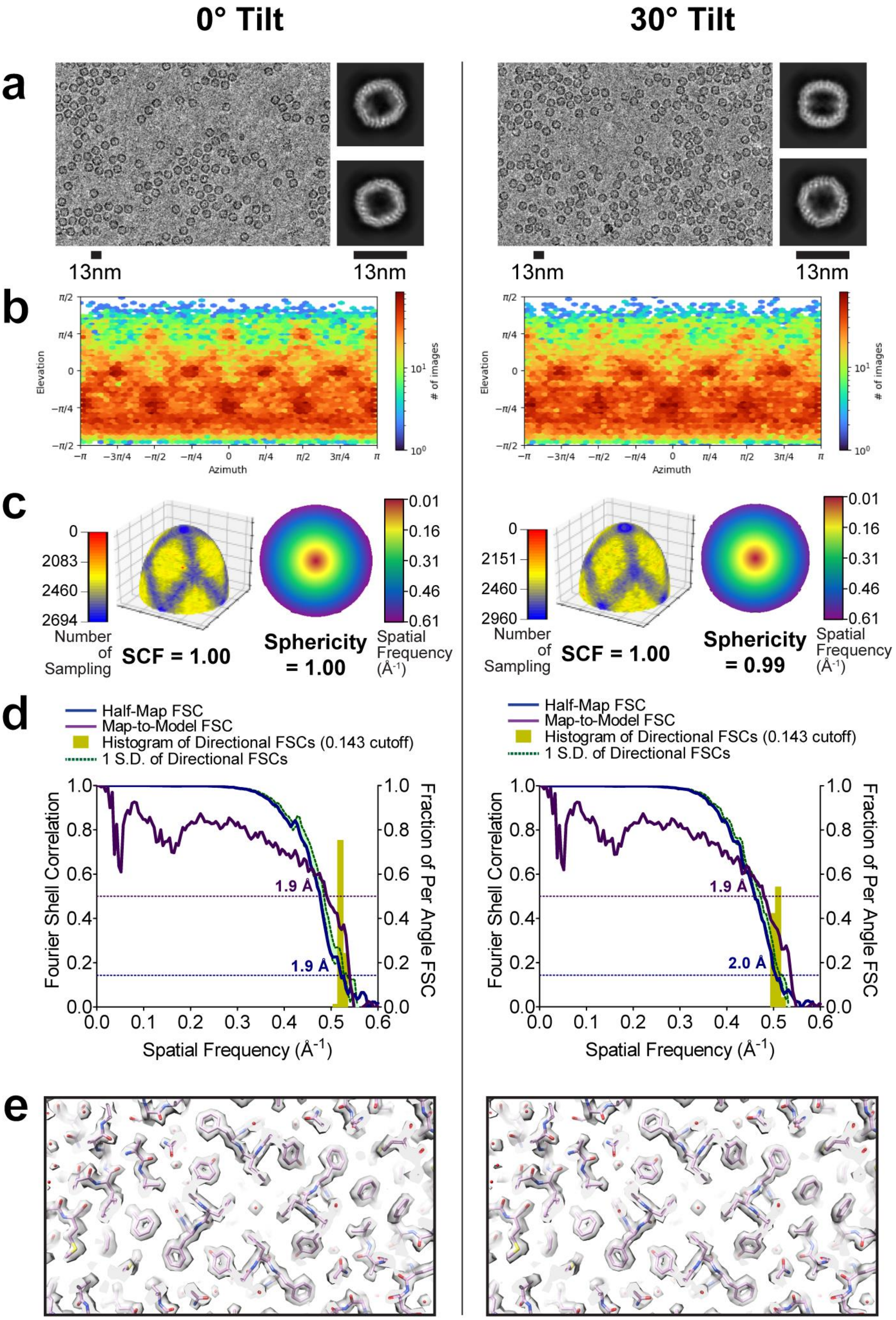
0° Tilt
30° Tilt
a
13nm
13nm
13nm
13nm
b
Elevation
Azimuth
# of images
c
Number of Sampling
SCF = 1.00
Sphericity = 1.00
Spatial Frequency (Å-1)
SCF = 1.00
Sphericity = 0.99
d
Half-Map FSC
Map-to-Model FSC
Histogram of Directional FSCs (0.143 cutoff)
1 S.D. of Directional FSCs
Fourier Shell Correlation
Fraction of Per Angle FSC
Spatial Frequency (Å-1)
1.9 Å
1.9 Å
1.9 Å
2.0 Å
e

Supplementary Figure 1 | High resolution reconstruction of apoferritin from 0° and 30° tilts. Using the updated processing strategy, apoferritin was collected without tilts and with a 30° stage tilt and equalized for defocus, with 64,000 particles used per tilt angle. (**a**) The micrographs at 1.1 μm defocus, 2D class averages, (**b**) final Euler angle distribution, sampling compensation factor (SCF) plot, (**c**) 3DFSC slice, (**d**) map-to-map and map-to-model FSC, and (**e**) map density with docked model are displayed for untilted (left) and 30° tilted (right) reconstructions.

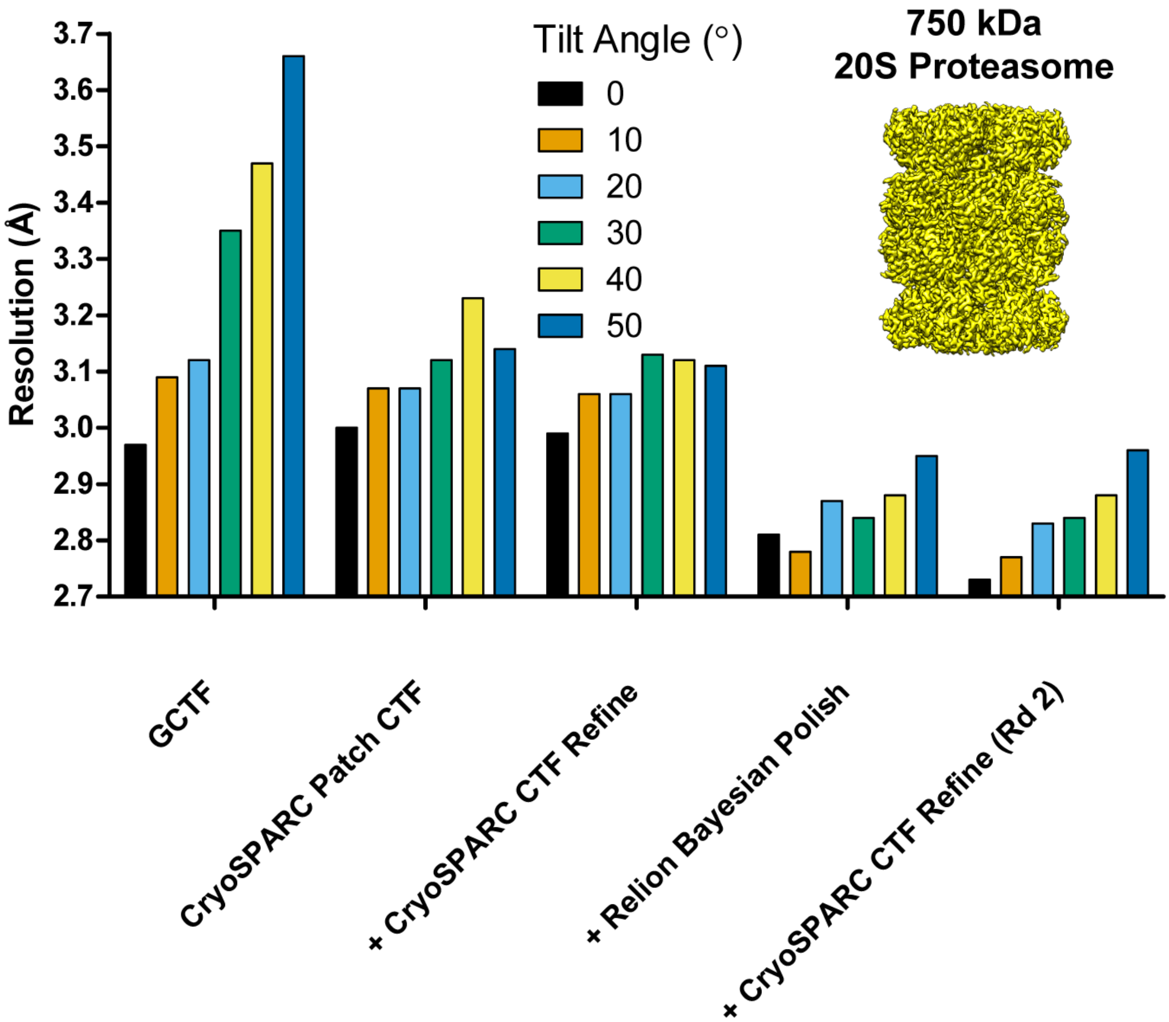

Supplementary Figure 2 | Improvement in resolution for the 20S proteasome at different tilt angles. The 20S proteasome cryo-EM data was collected at increasing stage tilt angles (0° black bars, 10° light orange bars, 20° light blue bars, 30° green bars, 40° yellow bars, 50° dark blue bars) and equalized for defocus, with 5,000 particles used per tilt angle. This figure follows the format of **Fig. 1**. The particles were processed using an identical workflow with global resolution at the end of each processing step plotted on the y-axis in Ångstroms (Å). (**a**) baseline processing strategy used in Tan et al[1]. (**b** to **e**) Individual steps were added to the workflow and improvements in resolution are plotted as lines. No further improvements were observed after the 2nd round of CTF refinement.

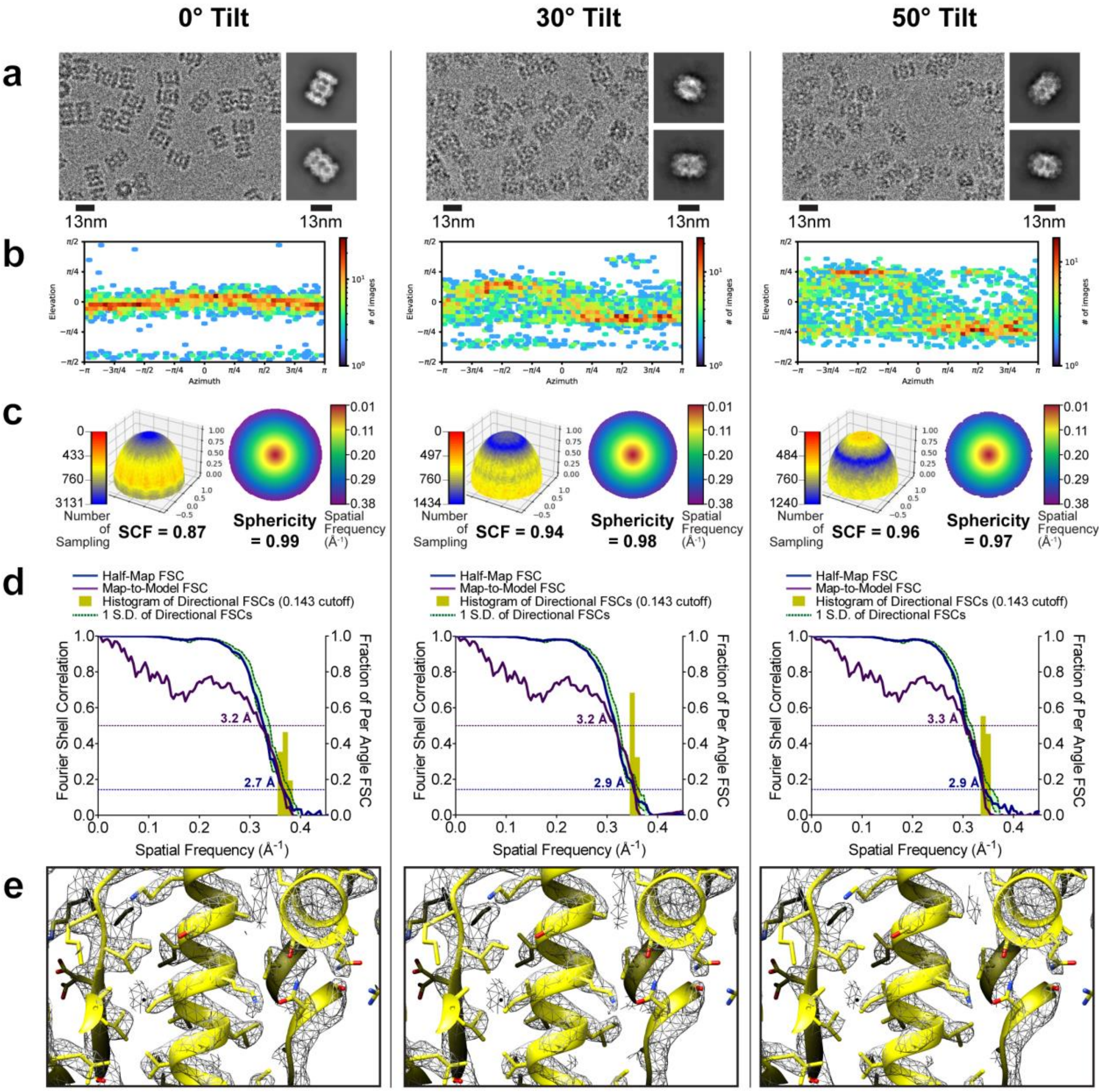

Supplementary Figure 3 | Evaluation metrics for the 20S proteasome at representative tilt angles. Evaluation metrics of the cryo-EM data and reconstructed maps for the processing strategy outlined in **Supplementary Fig. 2**. Results for untilted (left column), 30°-tilt (middle column) and 50°-tilt (right column) are compared. (**a**) Micrographs at 2 µm defocus, with corresponding 2D class averages at right. (**b**) Final Euler angle distribution. (**c**) Surface sampling plot with the sampling compensation factor (SCF) value below (left) and central slice through 3DFSC (right), coloured by resolution. (**d**) Half-map and map-to-model global FSC curves. (**e**) Map density with docked atomic model.

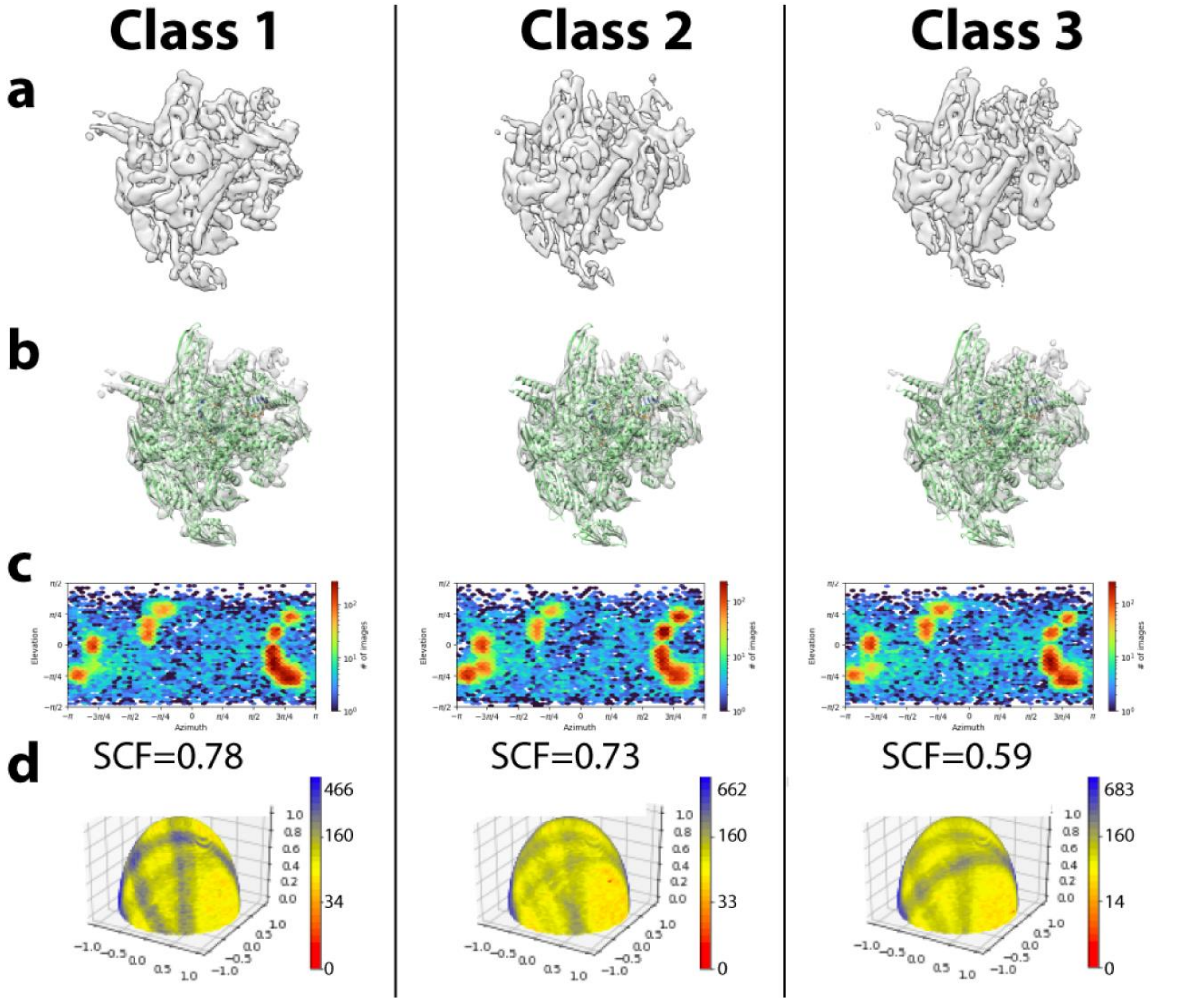

Supplementary Figure 4 | Cryo-EM maps derived from data collected at 51° stage tilt angle for RNAP. 85,340 particles are classified into 3 classes using Heterogeneous refinement in cryoSPARC, with 29,913 particles in class 1, 28,205 particles in class 2, and 27,222 particles in class 3. (**a**) Refined map without and (**b**) with a docked atomic model. (**c**) Euler angle distribution showing the particle orientations contributing to final map. (**d**) Surface sampling plot with calculated sampling compensation factor (SCF) values. The average SCF from the 3 maps is 0.70.

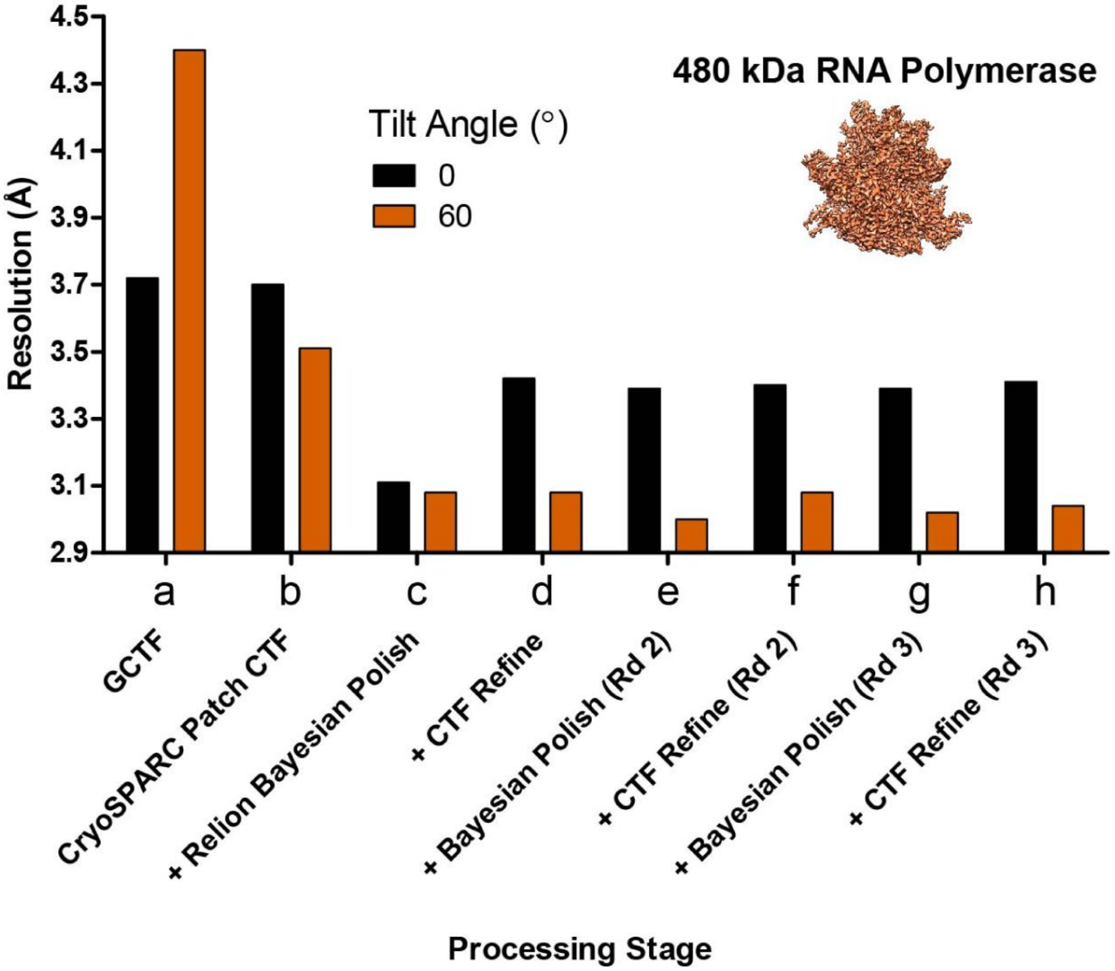

Supplementary Figure 5 | Improvement in resolution for RNAP collected at 0° and 60° tilt angles. RNAP cryo-EM data was collected at two tilt angles (0° black bars, 60° dark orange bars) and equalized for defocus, with 72,000 particles used per tilt angle. This figure follows the format of **Fig. 1**. The particles were processed using an identical workflow with global resolution at the end of each processing step plotted on the y-axis in Ångstroms (Å). (**a**) baseline processing strategy used in Tan et al[1]. (**b** to **h**) Individual steps were added to the workflow and improvements in resolution are plotted as lines.

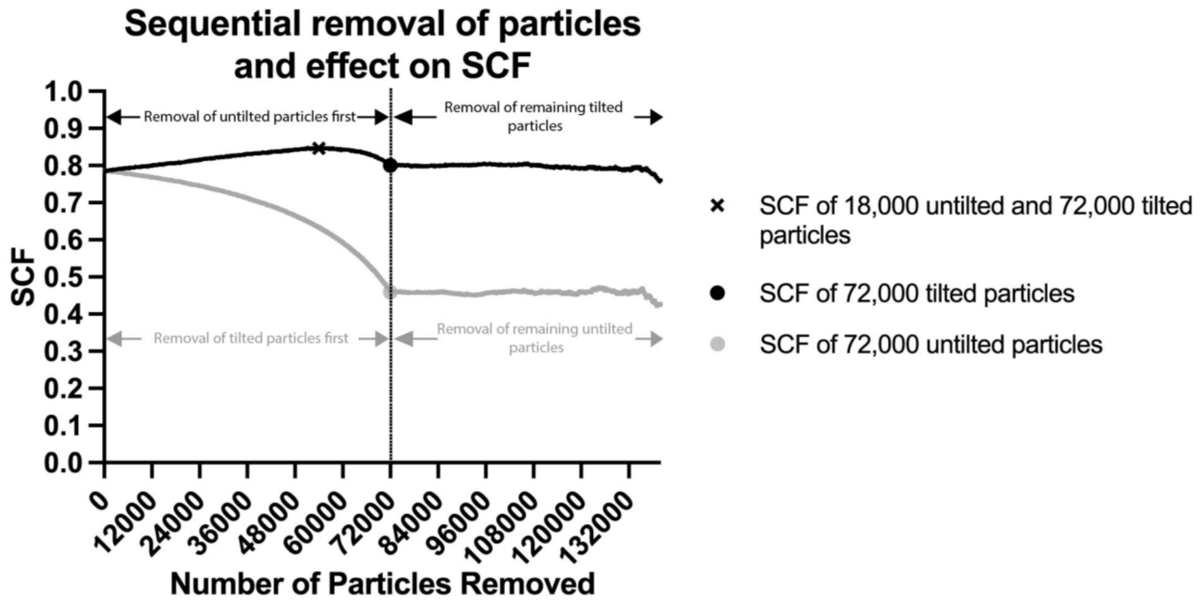

Supplementary Figure 6 | Effect of varying the number of tilted and untilted particles on the sampling compensation factor (SCF). From the total combined 144,000 particles (72,000 untilted + 72,000 tilted particles), we incrementally removed 100-particle subsets. The gray line corresponds to the removal of the tilted particles first, followed by the removal of the untilted particles. Grey filled circle corresponds to the SCF where only untilted particles remain. The black line corresponds to the removal of the untilted particles first, followed by the removal of the tilted particles. Black filled circle corresponds to the SCF where only tilted particles remain. Black cross corresponds to the maximum observed SCF in this analysis, which occurs when there are 18,000 untilted and 72,000 tilted particles. All particles were selected randomly from each stack, and the resulting SCF was calculated and plotted in 100-particle increments. In both datasets, when there are less than ~10,000 particles remaining, there are fluctuations in the SCF presumably due to sampling inhomogeneities and the lack of symmetry in RNAP.

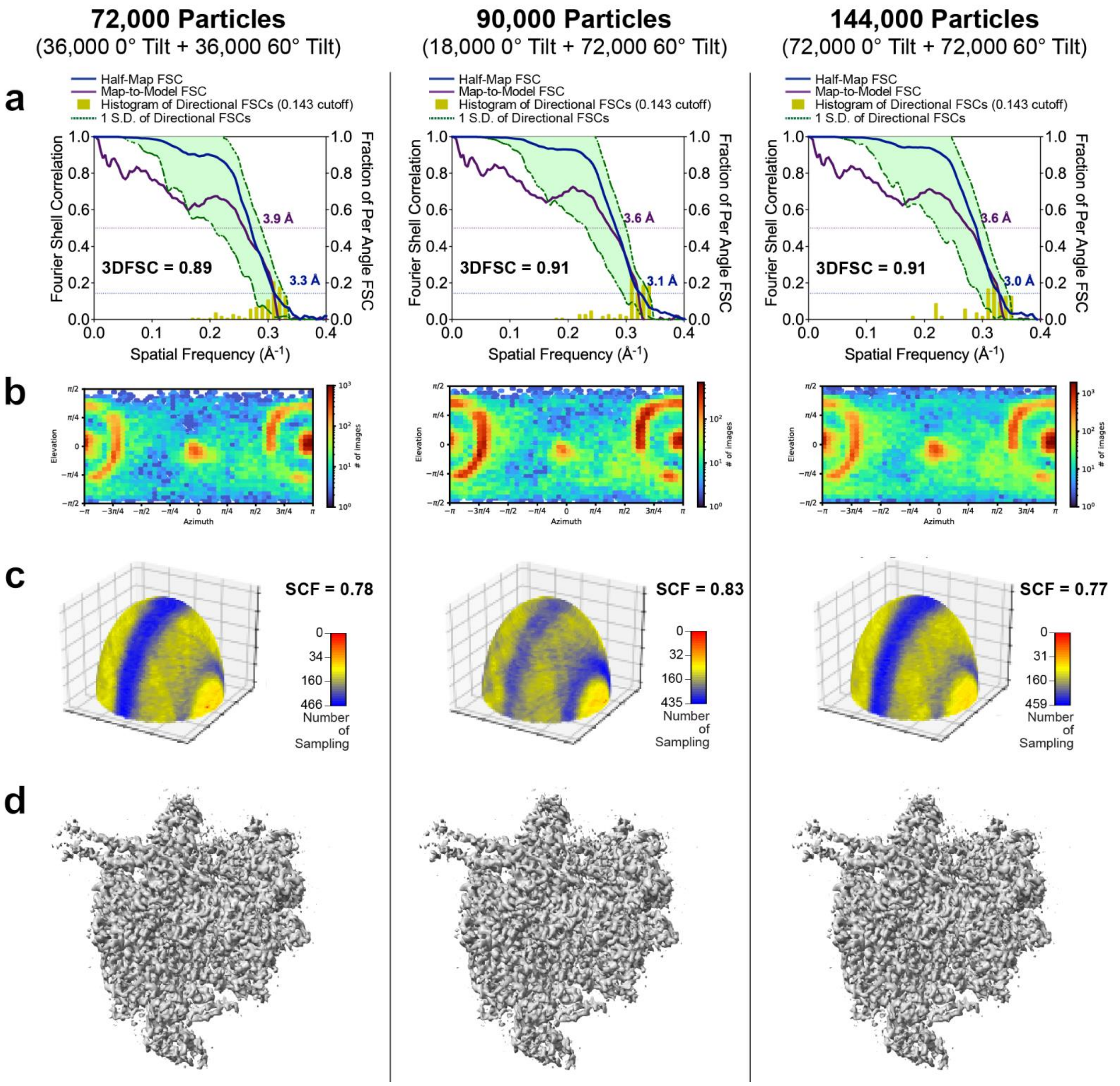

Supplementary Figure 7 | Combined refinement of 0° and 60° tilt angles RNAP dataset. RNAP cryo-EM data collected at two tilt angles (0°, 60°) were combined and refined together in three types of combinations: 36,000 untilted + 36,000 tilted particles, all randomly selected (left); 18,000 untilted + 72,000 tilted particles, in accordance to the titration analysis in **Supplementary Fig. 6** along the black curve (center); all 72,000 untilted + all 72,000 tilted particles, effectively doubling the stack size (right). The results of the refined reconstructions are shown, which include (**a**) the global, map-to-model and 3DFSCs, (**b**) Euler angle distribution, (**c**) the sampling distributions plotted on the surface of a sphere, along with the sampling compensation factor (SCF) values, and (**d**) the experimental density map.

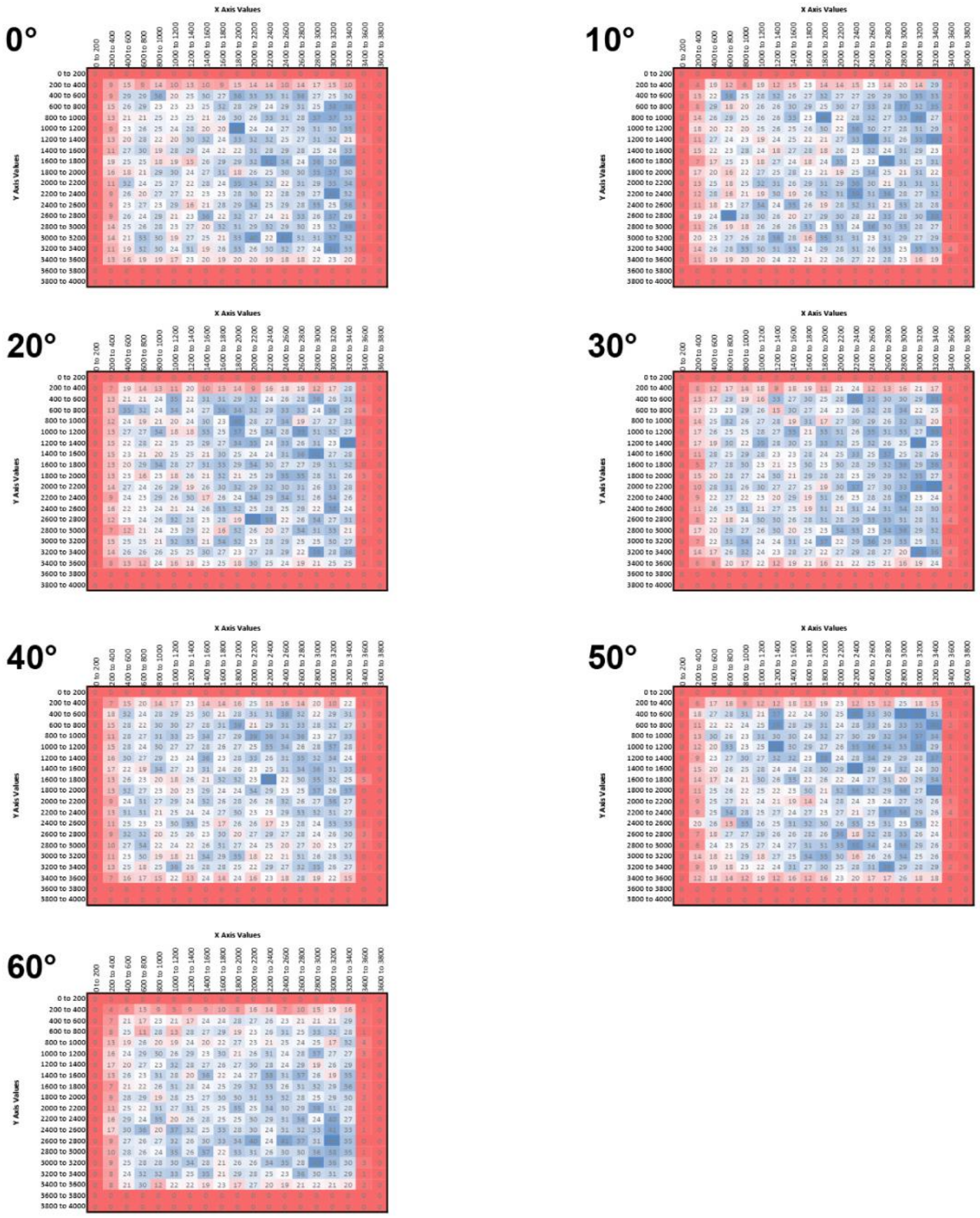

Supplementary Figure 8 | Histogram of particle coordinates for AAV2 at various tilt angles. AAV2 particle coordinates are plotted as a histogram of 200 pixel intervals for both X and Y axes, across various tilt angles.

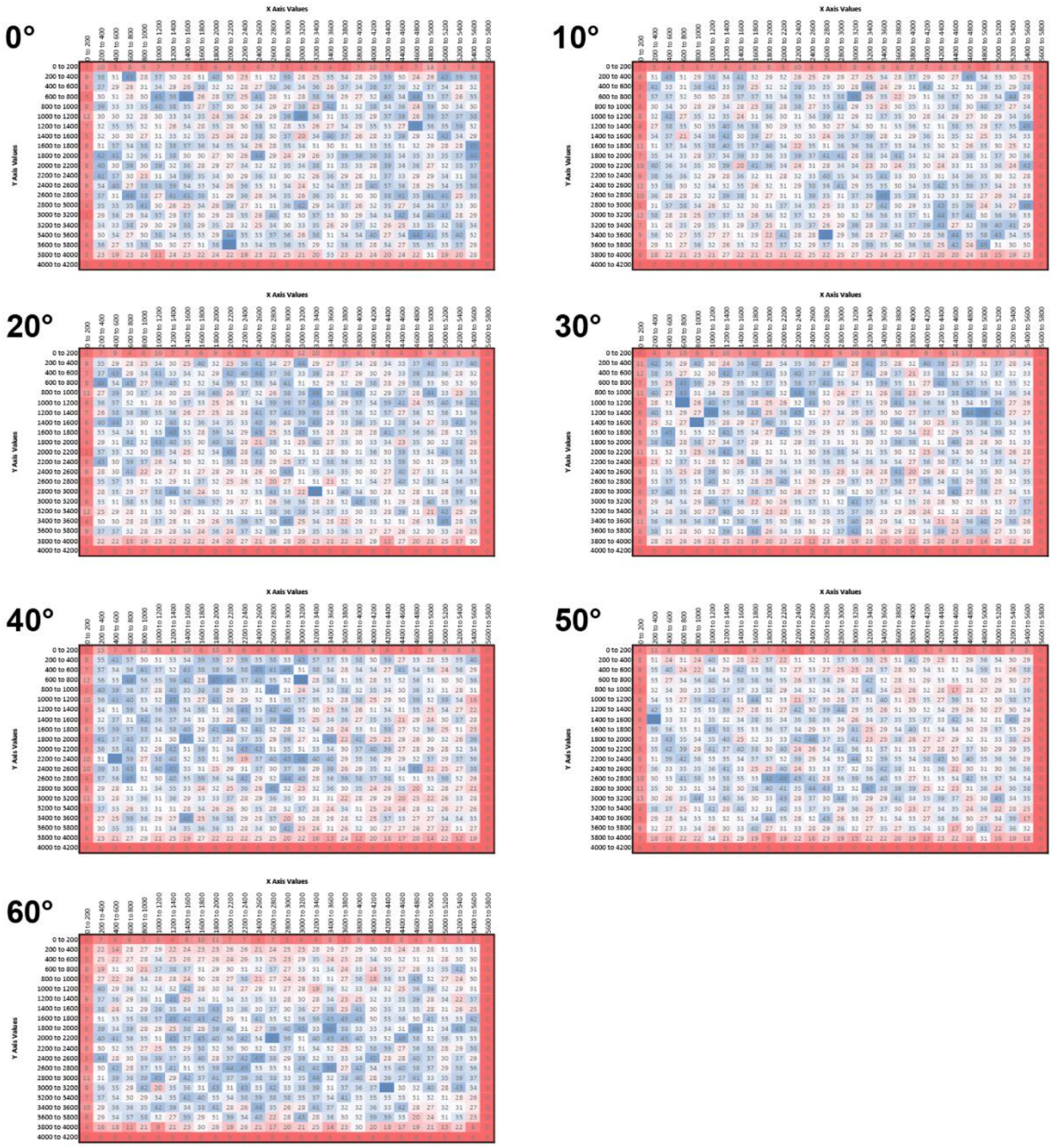

Supplementary Figure 9 | Histogram of particle coordinates for apoferritin at various tilt angles. Apoferritin particle coordinates are plotted as a histogram of 200 pixel intervals for both X and Y axes, across various tilt angles.

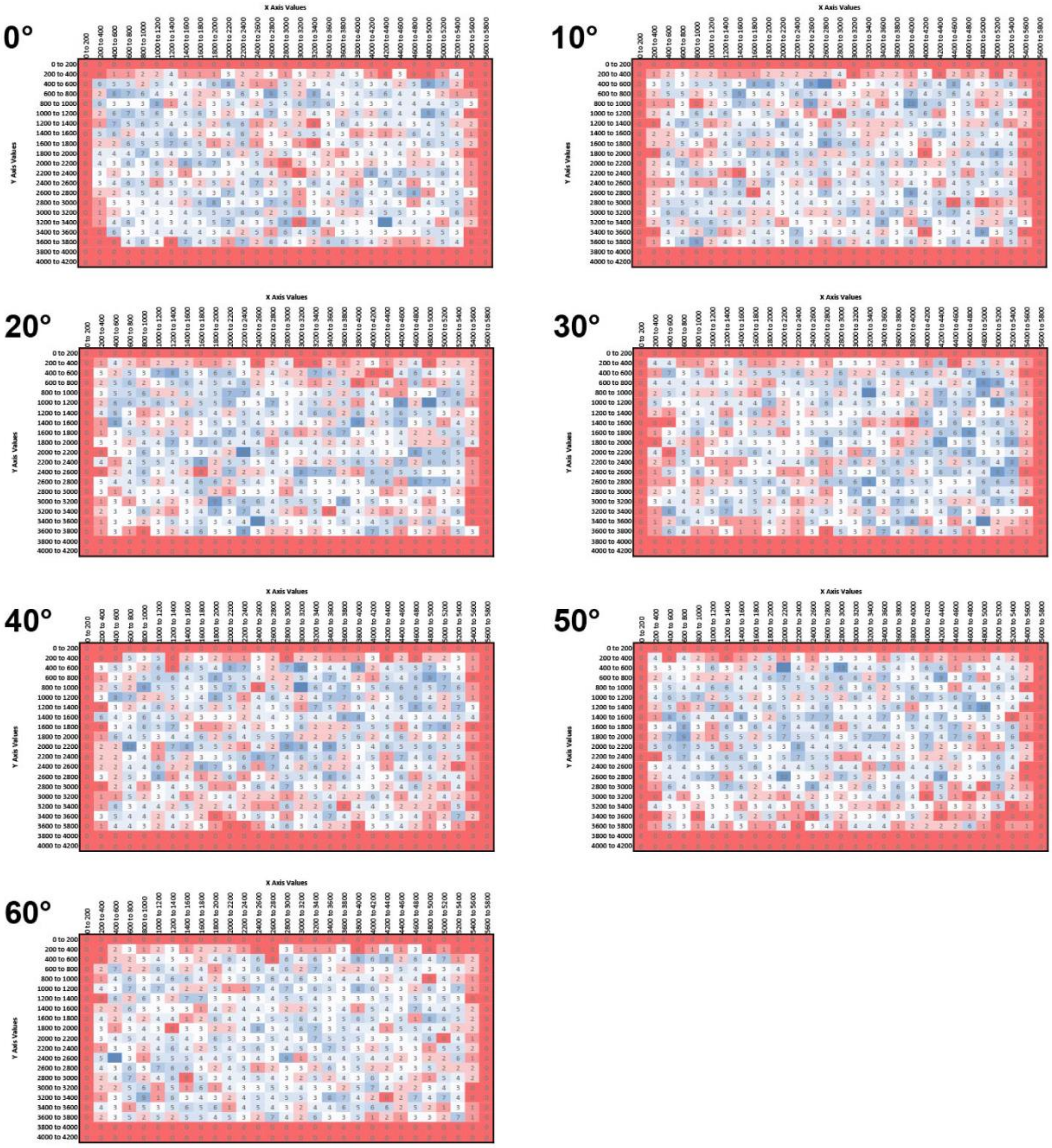

Supplementary Figure 10 | Histogram of particle coordinates for DPS at various tilt angles. DPS particle coordinates are plotted as a histogram of 200 pixel intervals for both X and Y axes, across various tilt angles.

Supplementary Table 1. Cryo-EM data collection for AAV2

EM data collection / processing							
**Tilt Angle (°)**	0	10	20	30	40	50	60
**Microscope**	FEI Titan Krios						
**Voltage (kV)**	300						
**Camera**	Gatan K2 Summit						
**Mode**	Counting						
**Energy filter slit width (eV)**	No energy filter						
**Defocus mean ± std (μm)**	1.5±0.3	1.5±0.3	1.5±0.3	1.4±0.4	1.4±0.4	1.5±0.4	1.6±0.4
**Exposure time (s)**	10						
**Number of fractions**	200						
**Exposure rate (e-/pixel/s)**	8.2	8.2	8.2	8.2	8.2	8.2	8.2
**Total exposure (e-/Å^2)**	78.0	78.0	78.0	78.0	78.0	78.0	78.0
**Pixel size (Å)**	1.026						
**Magnification**	29,000x						
**Number of micrographs**	166	180	194	168	185	181	155
**Defocus tilt angle (°)**	3±1	13±1	23±2	33±2	44±3	52±8	63±10
**Number of particles (in final map)**	7,000						
**Symmetry**	I						
**Resolution (global) (Å)**	2.1	2.1	2.1	2.2	2.1	2.1	2.2
**Directional Resolution Range (Å)**	2.1-2.1	2.1-2.1	2.1-2.1	2.1-2.2	2.1-2.2	2.1-2.1	2.1-2.2
**Sphericity of 3DFSC**	0.99	0.96	0.99	0.99	0.99	0.99	0.99
**SCF Value***	1.00	1.00	1.00	1.00	1.00	1.00	1.00
**Map B factor (Å^2)**	45.9	46.6	47.7	47.3	50.8	50.4	48.7
**Map-to-model FSC (global) (Å)**	2.1	2.1	2.1	2.1	2.1	2.1	2.2
**EMDB ID**	EMD-36766	EMD-36767	EMD-36768	EMD-36769	EMD-36770	EMD-36771	EMD-36772

*The sampling compensation factor (SCF) value is calculated as described (Baldwin and Lyumkis, 2020) and assumes that all orientations have been determined accurately.

Supplementary Table 2. Cryo-EM data collection statistics for apoferritin (Scripps Research)

EM data collection / processing							
**Tilt Angle (°)**	0	10	20	30	40	50	60
**Microscope**	FEI Titan Krios						
**Voltage (kV)**	300						
**Camera**	Gatan K3 BioQuantum						
**Mode**	Counting (CDS)						
**Energy filter slit width (eV)**	20						
**Defocus mean ± std (µm)**	1.4±0.3	1.4±0.4	1.4±0.4	1.3±0.3	1.4±0.4	1.4±0.4	1.5±0.3
**Exposure time (s)**	1.5						
**Number of fractions**	100						
**Exposure rate (e-/pixel/s)**	20.3	20.3	20.3	20.2	20.2	20.3	20.3
**Total exposure (e-/Å^2)**	44.2	44.2	44.1	44.0	44.0	44.1	44.1
**Pixel size (Å)**	0.830						
**Magnification**	105,000x						
**Number of micrographs**	74	87	79	100	120	91	86
**Defocus tilt angle (°)**	2±1	7±2	16±2	26±1	33±2	45±3	54±2
**Number of particles (in final map)**	17,000						
**Symmetry**	O						
**Resolution (global) (Å)**	2.2	2.3	2.3	2.2	2.4	2.3	2.5
**Directional Resolution Range (Å)**	2.1-2.3	2.2-2.3	2.3-2.4	2.2-2.3	2.3-2.4	2.2-2.3	2.4-2.5
**Sphericity of 3DFSC**	0.98	0.97	0.99	0.99	0.99	0.99	0.99
**SCF Value***	0.91	0.95	0.98	1.00	0.99	1.00	1.00
**Map B factor (Å^2)**	75.2	80.8	86.6	78.2	87.5	86.0	90.1
**Map-to-model FSC (global) (Å)**	2.2	2.3	2.4	2.3	2.4	2.4	2.5
**EMDB ID**	EMD-36807	EMD-36809	EMD-36810	EMD-36811	EMD-36812	EMD-36814	EMD-36813

*The sampling compensation factor (SCF) value is calculated as described (Baldwin and Lyumkis, 2020) and assumes that all orientations have been determined accurately.

Supplementary Table 3. Cryo-EM data collection statistics for apoferritin (Janelia Research Campus)

EM data collection / processing		
**Tilt Angle (°)**	0	30
**Microscope**	FEI Titan Krios	
**Voltage (kV)**	300	
**Camera**	Gatan K3 BioQuantum	
**Mode**	Counting (CDS)	
**Energy filter slit width (eV)**	20 eV	
**Defocus mean ± std (µm)**	0.86 ± 0.53	0.92 ± 0.47
**Exposure time (s)**	4.1	
**Number of fractions**	270	
**Exposure rate (e-/pixel/s)**	7.0	7.0
**Total exposure (e-/Å^2)**	41.2	41.2
**Pixel size (Å)**	0.83	
**Magnification**	105,000x	
**Number of micrographs**	229	403
**Defocus tilt angle (°)**	12±15	24±13
**Number of particles (in final map)**	64,000	
**Symmetry**	O	
**Resolution (global) (Å)**	1.93	1.99
**Directional Resolution Range (Å)**	1.9-2.0	1.9-2.0
**Sphericity of 3DFSC**	0.99	0.99
**SCF Value***	1.00	1.00
**Map B factor (Å^2)**	47.5	50.7
**Map-to-model FSC (global) (Å)**	1.9	1.9
**EMDB ID**	EMD-41230	EMD-41231

*The sampling compensation factor (SCF) value is calculated as described (Baldwin and Lyumkis, 2020) and assumes that all orientations have been determined accurately.

Supplementary Table 4. Cryo-EM data collection statistics for the 20S Proteasome

EM data collection / processing						
**Tilt Angle (°)**	0	10	20	30	40	50
**Microscope**	FEI Titan Krios					
**Voltage (kV)**	300					
**Camera**	Gatan K2 Summit					
**Mode**	Counting					
**Energy filter slit width (eV)**	No energy filter					
**Defocus mean ± std (µm)**	2.3±0.4	2.4±0.5	2.3±0.5	2.4±0.5	2.3±0.5	2.3±0.5
**Exposure time (s)**	14.00					
**Number of fractions**	70					
**Exposure rate (e-/pixel/s)**	7.7	7.7	7.7	7.7	7.7	7.7
**Total exposure (e-/Å²)**	93.8	93.8	93.8	93.8	93.8	93.8
**Pixel size (Å)**	1.07					
**Magnification**	22,500x					
**Number of micrographs**	82	35	46	108	92	57
**Defocus tilt angle (°)**	9±3	15±1	24±1	30±2	40±2	48±1
**Number of particles (in final map)**	5,000					
**Symmetry**	D7					
**Resolution (global) (Å)**	2.7	2.8	2.8	2.8	2.9	3.0
**Directional Resolution Range (Å)**	2.6-2.8	2.6-2.8	2.7-2.9	2.7-2.9	2.7-2.9	2.8-3.0
**Sphericity of 3DFSC**	0.99	0.99	0.99	0.98	0.99	0.97
**SCF Value***	0.87	0.89	0.93	0.94	0.96	0.96
**Map B factor (Å²)**	67.9	70.3	72.9	72.7	73.3	75.1
**Map-to-model FSC (global) (Å)**	3.2	3.1	3.2	3.2	3.3	3.3

*The sampling compensation factor (SCF) value is calculated as described (Baldwin and Lyumkis, 2020) and assumes that all orientations have been determined accurately.

Supplementary Table 5. Cryo-EM data collection statistics for DPS

EM data collection / processing							
**Tilt Angle (°)**	0	10	20	30	40	50	60
**Microscope**	FEI Titan Krios						
**Voltage (kV)**	300						
**Camera**	Gatan K3 BioQuantum						
**Mode**	Counting (No CDS)						
**Energy filter slit width (eV)**	20						
**Defocus mean ± std (µm)**	1.6±0.3	1.5±0.3	1.6±0.3	1.6±0.4	1.6±0.3	1.7±0.4	1.4±0.4
**Exposure time (s)**	2.54						
**Number of fractions**	195						
**Exposure rate (e-/pixel/s)**	16.6	16.6	16.6	16.6	16.6	16.6	16.6
**Total exposure (e-/Å^2)**	59.6	59.6	59.6	59.6	59.6	59.6	59.6
**Pixel size (Å)**	0.834						
**Magnification**	105,000x						
**Number of micrographs**	15	24	20	35	37	31	32
**Defocus tilt angle (°)**	7±2	14±4	19±3	29±4	37±3	48±3	56±3
**Number of particles (in final map)**	1,750						
**Symmetry**	T						
**Resolution (global) (Å)**	2.6	2.8	2.7	3.0	3.2	2.9	3.2
**Directional Resolution Range (Å)**	2.5-2.8	2.8-3.0	2.6-2.9	2.9-3.3	3.0-3.3	2.8-3.3	3.2-3.5
**Sphericity of 3DFSC**	0.94	0.98	0.99	0.99	0.98	0.97	0.97
**SCF Value***	0.95	0.98	0.99	0.99	0.99	0.99	0.99
**Map B factor (Å^2)**	24.8	35.6	27.2	36.6	34.6	30.0	46.5
**Map-to-model FSC (global) (Å)**	2.8	3.0	2.9	3.1	3.3	3.4	3.4
**EMDB ID**	EMD-36816	EMD-36817	EMD-36818	EMD-36819	EMD-36820	EMD-36821	EMD-36822

*The sampling compensation factor (SCF) value is calculated as described (Baldwin and Lyumkis, 2020) and assumes that all orientations have been determined accurately.

Supplementary Table 6. Cryo-EM data collection and modeling statistics for RNAP

EM data collection / processing		
**Tilt Angle (°)**	0	60
**Microscope**	FEI Titan Krios	
**Voltage (kV)**	300	
**Camera**	Gatan K3 BioQuantum	
**Mode**	Counting (CDS)	
**Energy filter slit width (eV)**	20	
**Defocus mean ± std (µm)**	1.3±0.4	1.8±0.7
**Exposure time (s)**	0.75	
**Number of fractions**	50	
**Exposure rate (e-/pixel/s)**	31.4	35.6
**Total exposure (e-/Å^2)**	34.2	38.8
**Pixel size (Å)**	0.830	
**Magnification**	105,000x	
**Number of micrographs**	619	527
**Defocus tilt angle (°)**	5±2	52±5
**Number of particles (in final map)**	72,000	
**Symmetry**	C1	
**Resolution (global) (Å)**	3.5	3.0
**Directional Resolution Range (Å)**	3.2-12.3	2.9-5.2
**Sphericity of 3DFSC**	0.76	0.90
**SCF Value***	0.45	0.78
**Map B factor (Å^2)**	-47.7	-75.9
**Map-to-model FSC (global) (Å)**	6.8	3.5
**EMDB ID**	-	41695
**PDB ID**	-	8TXO
**Initial model used (PDB code)**	-	8SY7
**Model resolution (Å)**	-	3.4
**FSC threshold**	-	0.5
**Model resolution range (Å)**	-	2.98-3.40
**Model composition**		
**Non-hydrogen atoms**	-	19,794
**Protein**	-	2,614
**Ligands**	-	5
**B factors (Å^2)**		
**Protein**	-	80.94
**Nucleotide**	-	132.89
**Ligand**	-	80.91
**R.m.s deviations**		
**Bond lengths (Å)**	-	0.009
**Bond angles (°)**	-	0.902
**Validation**		

**MolProbity score**	-	1.76
**Clashscore**	-	9.88
**Poor rotamers (%)**	-	0.00
**Ramachandran plot**		
**Favored (%)**	-	96.32
**Allowed (%)**	-	3.68
**Disallowed (%)**	-	0.00

*The sampling compensation factor (SCF) value is calculated as described (Baldwin and Lyumkis, 2020) and assumes that all orientations have been determined accurately.

Supplementary Note 1

For reconstructions from 0° data, the Fourier shell correlation curves indicated that the map was initially resolved to nominal resolution of 3.7 Å. This resolution improved to 3.1 Å after implementing the Bayesian polishing routine, but then worsened to and stabilized at 3.4 Å in subsequent stages of processing. The fluctuations in resolution (**Supplementary Fig. 5**) indicate several issues: the nominal resolution values of ~3–4 Å are inconsistent with the features evident within the maps; the fluctuations in nominal resolution are inconsistent with the more systematic improvements that we observe in all other datasets, and with the expected behavior of refinement protocols. We attribute these inconsistencies to the pathologically preferential orientation distribution of RNAP, and as a result, to missing data in the reconstructed map. As previously shown, a single top-like orientation distribution for a particle leads to heavily undersampled and/or missing data in Fourier space within the reconstruction[2]. This undersampling is present in both half-maps that are used to compute the FSC and the voxels may be correlated and reinforced during refinement, skewing the calculation of the global FSC. There is also a second possible issue that was previously described[2], wherein the FSC curve becomes inflated if there are zeros present in the transform, e.g. due to missing or sparsely assigned orientations (in practice, we do not know whether the orientations assigned into sparsely populated regions of Euler space are true positives). For these reasons, in cases characterized by severe preferred orientation, the global half-map FSC may not be useful in judging the improvement in map quality.

Supplementary Note 2

We wanted to assess how the SCF changes when different numbers of untilted or tilted particles are used for the reconstruction. To test this, we performed two types of titration experiments using RNAP: (Experiment 1) Incrementally remove particles from the tilted stack first, at 100-particle intervals, followed by incremental removal of the 72,000 untilted particles; (Experiment 2) the converse experiment, i.e. first remove the untilted particles, then remove the tilted particles. In experiment 1, the SCF systematically decreases and approaches that of the reconstruction from the untilted stack, which is SCF=0.45 (**Supplemental Fig. 6**, gray line). This titration served as a positive control and indicated that, as expected, the tilted particles are the ones that contribute to the isotropic, high-resolution map. Intriguingly, when we removed the untilted particles first in experiment 2, we noticed that the SCF gradually increases to ~0.83, but then starts to drop back to its baseline value of ~0.78 (**Supplemental Fig. 6**, black line). In other words, there appeared to be a narrow window within which combining some small amount of untilted images with primarily tilted images could make sense, based solely on the SCF value. The peak SCF of ~0.83 occurred when there were ~90,000 particles in the stack (i.e. 72000 tilted + ~18000 tilted particles) (**Supplementary Fig. 6**, black cross).

We next assessed the effects of combining untilted and tilted particle stacks by refining and reconstructing three datasets: (i) the combination of randomly selected 36,000 particles from untilted and tilted datasets each, yielding a 72,000-particle stack that is directly comparable to the results in **Figure 8**; (ii) a 90,000 particles stack that includes 72,000 particles from the 60°-tilted dataset and 18,000 particles from the untilted dataset, based on the titration experiment described above and shown in **Supplementary Fig. 6**; (iii) all particles combined, yielding a 144,000-particle stack and effectively doubling the particle count. The reconstructions and validation metrics are shown in **Supplementary Fig. 7**. In experiment (i), we derived an inferior map by all metrics in comparison to the map derived from 60° tilted images alone (compare with **Figure 8**). Interestingly, in experiment (ii) we did not notice any tangible improvements in comparison to reconstructions from the tilted images alone. This is likely because a small improvement in SSNR (and by extension, the SCF), such as from 0.79→0.83, will yield an even smaller improvement in the FSC, according to its definition[2,3]. Thus, the fluctuations in the FSC curves are likely within the noise limits of detection. In experiment (iii), the resulting map was again comparable in quality to the map from tilted images alone. We conclude, as previously suggested[1], that combining tilted and untilted images will either lead to an inferior map or will not benefit the experimentalist under most experimental conditions; signal is contributed by the tilted particles, whereas the untilted particles hyper-sample around one Fourier plane. However, we cannot exclude the possibility outright that combining tilted and untilted particles is ineffective under all conditions, as there may still be benefits, e.g., when the orientation distribution is less pathological, when only a small number of untilted images are included, or if the tilted images are inferior in quality.

Supplementary References

1 Tan, Y. Z. *et al.* Addressing preferred specimen orientation in single-particle cryo-EM through tilting. *Nature methods* **14**, 793-796 (2017).

2 Baldwin, P. R. & Lyumkis, D. Non-uniformity of projection distributions attenuates resolution in Cryo-EM. *Progress in biophysics and molecular biology* **150**, 160-183 (2020).

3 Baldwin, P. R. & Lyumkis, D. Tools for visualizing and analyzing Fourier space sampling in Cryo-EM. *Progress in Biophysics and Molecular Biology* **160**, 53-65 (2021).
