# Supplementary material for: Overcoming resolution attenuation during tilted cryo-EM data collection
Source: Nat Commun. 2024 Jan 9;15:389. doi: 10.1038/s41467-023-44555-7 (PMC10776679; doi:10.1038/s41467-023-44555-7)
Supplement: Supplementary file 3 — Description of Additional Supplementary Files [file 41467_2023_44555_MOESM3_ESM.pdf]

Description of Additional Supplementary Files

File Name: Supplementary Movie 1
Description: Tomographic reconstruction of a tilt-series showing RNAP particles at the air-water interface. For colour scheme and description of tilt-series, refer to Fig. 7.
