# Supplementary material for: Overcoming resolution attenuation during tilted cryo-EM data collection
Source: Nat Commun. 2024 Jan 9;15:389. doi: 10.1038/s41467-023-44555-7 (PMC10776679; doi:10.1038/s41467-023-44555-7)
Supplement: Supplementary file 5 — Reporting Summary [file 41467_2023_44555_MOESM5_ESM.pdf]

nature portfolio

Corresponding author(s): Dmitry Lyumkis, Yong Zi Tan

Last updated by author(s): Dec 6, 2023

Reporting Summary

Nature Portfolio wishes to improve the reproducibility of the work that we publish. This form provides structure for consistency and transparency in reporting. For further information on Nature Portfolio policies, see our Editorial Policies and the Editorial Policy Checklist.

Statistics

For all statistical analyses, confirm that the following items are present in the figure legend, table legend, main text, or Methods section.

n/a	Confirmed	
☐	☒	The exact sample size (*n*) for each experimental group/condition, given as a discrete number and unit of measurement
☒	☐	A statement on whether measurements were taken from distinct samples or whether the same sample was measured repeatedly
☒	☐	The statistical test(s) used AND whether they are one- or two-sided<br>*Only common tests should be described solely by name; describe more complex techniques in the Methods section.*
☒	☐	A description of all covariates tested
☒	☐	A description of any assumptions or corrections, such as tests of normality and adjustment for multiple comparisons
☐	☒	A full description of the statistical parameters including central tendency (e.g. means) or other basic estimates (e.g. regression coefficient) AND variation (e.g. standard deviation) or associated estimates of uncertainty (e.g. confidence intervals)
☒	☐	For null hypothesis testing, the test statistic (e.g. *F*, *t*, *r*) with confidence intervals, effect sizes, degrees of freedom and *P* value noted<br>*Give P values as exact values whenever suitable.*
☒	☐	For Bayesian analysis, information on the choice of priors and Markov chain Monte Carlo settings
☒	☐	For hierarchical and complex designs, identification of the appropriate level for tests and full reporting of outcomes
☒	☐	Estimates of effect sizes (e.g. Cohen's *d*, Pearson's *r*), indicating how they were calculated

Our web collection on statistics for biologists contains articles on many of the points above.

Software and code

Policy information about availability of computer code

Data collection	Serial EM- Data collection at Janelia Research Campus and Pacific Northwest Center for CryoEM<br>Leginon- Data collection at Scripps Research and New York Structural Biology Consortium
Data analysis	Relion 3.0 and 3.1- For data processing of single-particle cryo-EM and cryo-ET<br>UCSF MotionCor2<br>Cryosparc v4.2 .l- For data processing of single-particle cryo-EM<br>WARP- For data processing of cryo-ET<br>I MOD- For data processing of cryo-ET<br>EMAN2- For data processing of single particle cryo-EM and cryo-ET<br>UCSF ChimeraX 1.6.1- For data visualization, generation of 3DFSC surface slices for directional resolution and rigid body fitting of atomic model into experimental EM map.<br>SCF- For data analysis, this is available via GitHub https://github. com/LyumkisLab/SamplingGui<br>3DFSC- For estimation of directional resolution<br>Appion- For data processing of single-particle cryo-EM<br>CTFFind4 and GCTF- For estimation of contrast transfer function of summed images<br>Amira- For data processing of cryo-ET<br>PyTom- For data processing of cryo-ET.

For manuscripts utilizing custom algorithms or software that are central to the research but not yet described in published literature, software must be made available to editors and reviewers. We strongly encourage code deposition in a community repository (e.g. GitHub). See the Nature Portfolio guidelines for submitting code & software for further information.

Data

Policy information about availability of data

All manuscripts must include a data availability statement. This statement should provide the following information, where applicable:

- Accession codes, unique identifiers, or web links for publicly available datasets
- A description of any restrictions on data availability
- For clinical datasets or third party data, please ensure that the statement adheres to our policy

All raw movie frames, micrographs, the particle stack, and relevant metadata files for the untilted and tilted datasets, generated in this study, are deposited in the EMPIAR database as EMPIAR-11791 (AAV2), EMPIAR-11792 (Apoferritin), EMPIAR-11796 (DPS) and EMPIAR-11797 (RNAP). The electron potential maps of AAV2 at various tilts, generated in this study, are deposited into the electron microscopy databank as EMD-36766 (0°), EMD-36767 (10°), EMD-36768 (20°), EMD-36769 (30°), EMD-36770 (40°), EMD-36771 (50°) and EMD-36772 (60°). The electron potential maps of apoferritin (Scripps Research) at various tilts, generated in this study, are deposited into the electron microscopy databank as EMD-36807 (0°), EMD-36809 (10°), EMD-36810 (20°), EMD-36811 (30°), EMD-36812 (40°), EMD-36814 (50°) and EMD-36813 (60°). The electron potential maps of apoferritin (Janelia Research Campus) at various tilts, generated in this study are deposited into the electron microscopy databank as EMD-41230 (0°) and EMD-41231 (30°). The electron potential maps of DPS at various tilts, generated in this study, are deposited into the electron microscopy databank as EMD-36816 (0°), EMD-36817 (10°), EMD-36818 (20°), EMD-36819 (30°), EMD-36820 (40°), EMD-36821 (50°) and EMD-36822 (60°). The model for RNAP, generated in this study, has been deposited into the PDB as PDB ID-8TXO and the electron potential map for the 60° tilted dataset, generated in this study, is deposited into the electron microscopy databank as EMD-41695. Other data are available from the corresponding authors upon request. The model for AAV2, not generated in this study, is available in the PDB as PDB ID-6E9D. The model for apoferritin, not generated in this study, is available in the PDB as PDB ID-7A6A. The model for Proteasome, not generated in this study, is available in the PDB as PDB ID-1YAR. The model for DPS, not generated in this study, is available in the PDB as PDB ID-6GCM.

Human research participants

Policy information about studies involving human research participants and Sex and Gender in Research.

Reporting on sex and gender	N/A
Population characteristics	N/A
Recruitment	N/A
Ethics oversight	N/A

Note that full information on the approval of the study protocol must also be provided in the manuscript.

Field-specific reporting

Please select the one below that is the best fit for your research. If you are not sure, read the appropriate sections before making your selection.

[x] Life sciences [ ] Behavioural & social sciences [ ] Ecological, evolutionary & environmental sciences

For a reference copy of the document with all sections, see nature.com/documents/nr-reporting-summary-flat.pdf

Life sciences study design

All studies must disclose on these points even when the disclosure is negative.

Sample size	Sample size pertaining to final particle numbers contributing to the final reconstruction of cryo-EM maps were selected based on quality of global and directional resolution. Once a sufficiently high-resolution reconstruction was obtained for a given specimen at a specific tilt angle, thereafter sample size was controlled internally by selecting equal number of particles for all tilt angles for respective specimen.
Data exclusions	Recorded movies of poor quality and false-positive picks during particle packing were excluded during the analysis.
Replication	All of the data collection and processing experiments were performed once without any repetition.
Randomization	To obtain equal number of comparable particles across the different tilt angles, we computationally equalized the datasets by selecting equal number of particles randomly with similar defocus spreads.
Blinding	Blinding is not relevant to the study since cryo-EM imaging conditions and data processing steps are not expected to be affected whether or not investigators are blinded.

Reporting for specific materials, systems and methods

We require information from authors about some types of materials, experimental systems and methods used in many studies. Here, indicate whether each material, system or method listed is relevant to your study. If you are not sure if a list item applies to your research, read the appropriate section before selecting a response.

Materials & experimental systems

n/a	Involved in the study
☒	☐ Antibodies
☐	☒ Eukaryotic cell lines
☒	☐ Palaeontology and archaeology
☒	☐ Animals and other organisms
☒	☐ Clinical data
☒	☐ Dual use research of concern

Methods

n/a	Involved in the study
☒	☐ ChIP-seq
☒	☐ Flow cytometry
☒	☐ MRI-based neuroimaging

Eukaryotic cell lines

Policy information about cell lines and Sex and Gender in Research

Cell line source(s)	Sf9 insect cell line (ATCC) is a clonal isolate derived from Spodoptera frugiperda.
Authentication	Cell lines were not authenticated by authors.
Mycoplasma contamination	Cell lines obtained from Thermo Fisher are negative for mycoplasma contamination. No in-house test for mycoplasma was performed.
Commonly misidentified lines (See ICLAC register)	N/A
